# Transcriptomic and phosphoproteomic profiling and metabolite analyses reveal the mechanism of NaHCO_3_-induced organic acid secretion in grapevine roots

**DOI:** 10.1186/s12870-019-1990-9

**Published:** 2019-09-03

**Authors:** Guangqing Xiang, Wanyun Ma, Shiwei Gao, Zhongxin Jin, Qianyu Yue, Yuxin Yao

**Affiliations:** State Key Laboratory of Crop Biology, Collaborative Innovation Center of Fruit & Vegetable Quality and Efficient Production, College of Horticulture Science and Engineering, Shandong Agricultural University, Tai-An, 271018 Shandong China

**Keywords:** Grapevine, Transcriptome, Phosphoproteome, Organic acid secretion, Alkali stress

## Abstract

**Background:**

Organic acid secretion is a widespread physiological response of plants to alkalinity. However, the characteristics and underlying mechanism of the alkali-induced secretion of organic acids are poorly understood.

**Results:**

Oxalate was the main organic acid synthesized and secreted in grapevine (a hybrid of *Vitis amurensis*, *V. berlandieri* and *V. riparia*) roots, while acetate synthesis and malate secretion were also promoted under NaHCO_3_ stress. NaHCO_3_ stress enhanced the H^+^ efflux rate of grapevine roots, which is related to the plasma membrane H^+^-ATPase activity. Transcriptomic profiling revealed that carbohydrate metabolism was the most significantly altered biological process under NaHCO_3_ stress; a total of seven genes related to organic acid metabolism were significantly altered, including two phosphoenolpyruvate carboxylases and phosphoenolpyruvate carboxylase kinases. Additionally, the expression levels of five *ATP-binding cassette transporters*, particularly *ATP-binding cassette B19*, and two *Al-activated malate transporter 2 s* were substantially upregulated by NaHCO_3_ stress. Phosphoproteomic profiling demonstrated that the altered phosphoproteins were primarily related to binding, catalytic activity and transporter activity in the context of their molecular functions. The phosphorylation levels of phosphoenolpyruvate carboxylase 3, two plasma membrane H^+^-ATPases 4 and ATP-binding cassette B19 and pleiotropic drug resistance 12 were significantly increased. Additionally, the inhibition of ethylene synthesis and perception completely blocked NaHCO_3_-induced organic acid secretion, while the inhibition of indoleacetic acid synthesis reduced NaHCO_3_-induced organic acid secretion.

**Conclusions:**

Our results demonstrated that oxalate was the main organic acid produced under alkali stress and revealed the necessity of ethylene in mediating organic acid secretion. Additionally, we further identified several candidate genes and phosphoproteins responsible for organic acid metabolism and secretion.

**Electronic supplementary material:**

The online version of this article (10.1186/s12870-019-1990-9) contains supplementary material, which is available to authorized users.

## Background

Soil alkalinity is an important environmental problem, and alkali stress primarily caused by NaHCO_3_ and Na_2_CO_3_ severely affects crop growth and development in more than 434 million hectares of land worldwide [1]. Compared with neutral salt stress, alkali stress not only causes osmotic stress and ion injury but also leads to high pH injury [2, 3]; therefore, alkali stress is more destructive than salt stress [4]. However, in contrast to the extensive studies on plant salt tolerance, much less attention has been focused on exploring the mechanisms underlying alkali stress tolerance.

Under alkali stress, plants must regulate intracellular pH and the pH outside roots to maintain root functions. Organic acids play a key role in regulating the cell and rhizosphere pH levels. It was reported that organic acid metabolism is closely correlated with alkali stress tolerance [4]. *Puccinellia tenuiflora* roots accumulate and secrete citric acid into the rhizosphere in response to alkali stress [5]. HCO_3_^−^ induces the production of malate, succinate, and citrate in rice roots [6] and malate in maize roots [7]. Additionally, organic acids have been reported to function in plant adaptation to other abiotic stresses. For example, cotton and alfalfa accumulate more citric acid under drought and salt stresses, respectively [8, 9]. Plants accumulate organic acids in cells and secrete them into rhizospheres when facing Al, P and Cd toxicity [10–12]. With regard to the mechanisms of alkali-induced organic acid synthesis and secretion, some key enzymes involved in organic acid metabolism, such as citrate synthase, malate synthase and isocitrate lyase, have been suggested to determine alkali stress tolerance [13]. Plasma membrane H^+^-ATPases, such as *Arabidopsis* AHA2 and AHA7, are necessary for proton secretion for plant tolerance to alkali stress [11, 14]. Additionally, some studies have demonstrated that ethylene and auxin have roles in the regulation of root H^+^ secretion and alkali stress by regulating H^+^-ATPase [15, 16].

To date, the pathways of organic acid metabolism in the cell and extrusion out of the cell under alkali stress remain largely unknown in grapevine. Genome-scale analysis of gene expression profiles is a powerful method to reveal plant abiotic stress tolerance mechanisms [17, 18]. For example, transcriptome profiling reveals the genetic basis of alkalinity tolerance in wheat [18] and gene networks responsive to NaHCO_3_ stress in *Tamarix hispida* [17]. Quantitative and comprehensive insights into the mRNA transcriptome will contribute to unraveling the key mechanism of organic acid secretion in response to alkali treatment. On the other hand, phosphorylation represents one of the most important posttranslational modification functions in diverse biological pathways. The activities of several proteins related to organic acid synthesis and extrusion, such as phosphoenolpyruvate carboxylase (PEPC) and H^+^-ATPase [19, 20], have been shown to be regulated by phosphorylation. An analysis of the comprehensive phosphorylation modifications induced by alkali stress is needed to better understand the process of organic acid secretion under alkali stress.

Grapevines are an economically important fruit crop worldwide that grow as deep-rooted perennial plants, and their growth and fruit quality are largely influenced by soil alkalinity and salinity. The majority of commercial rootstocks and varieties used in viticulture are moderately sensitive to alkali stress. However, the candidate rootstock cultivar A15 possesses strong alkali tolerance and is a suitable material for identifying alkali tolerance-associated genes. Additionally, A15 was identified to have an higher capacity to secrete organic acids than other grapevine rootstocks with moderate or weak tolerance to alkali stress [21]. The present study determined the accumulation and secretion of organic acids and H^+^ efflux at different time points after NaHCO_3_ treatment. Thereafter, the key genes and phosphoproteins involved in the above processes were identified using transcriptomic and phosphoproteomic analyses. Additionally, the roles of ethylene and indoleacetic acid (IAA) in mediating organic acid secretion under alkali conditions were evaluated. The results provide valuable information for dissecting the metabolism and regulatory pathways of alkali stress tolerance in grapevine roots.

## Results

### Effects of NaHCO_3_ on the synthesis and secretion of organic acids in grapevine roots

**Fig. 1:**
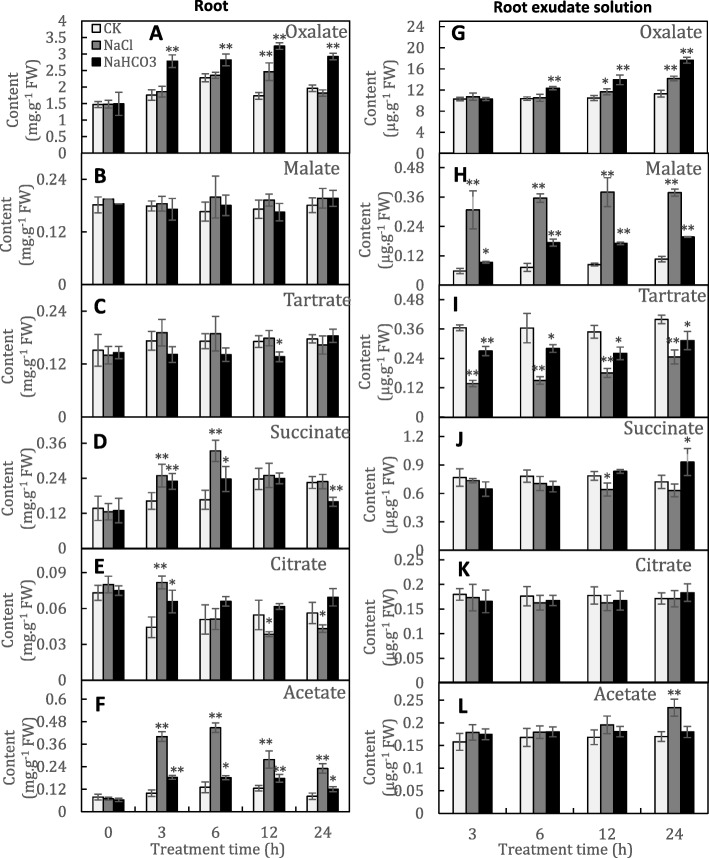
The organic acid content in grapevine roots (**a**-**f**) and their culture solution (**g**-**l**) under nonstress, salt-stress and alkali-stress conditions. The organic acid content in the root exudate solution was expressed using the ratio of the content of organic acids in the root exudate solution to the root weight to reflect root secretion capacity. Values represent the means ± SD of three replicates. * Significant difference, *P* < 0.05; ** highly significant difference, *P* < 0.01

A total of six organic acids were identified, oxalate, malate, tartrate, succinate, citrate and acetate. Oxalate was the main organic acid and accounted for 69.5–71.3% of the sum of the six organic acids in the control roots. In contrast, the five other organic acids, particularly citrate, showed relatively low concentrations (Fig. 1a-f). Similar differences in organic acid concentrations were also found in the culture solution (Fig. 1g-l); therefore, oxalate was the main organic acid synthesized and secreted by grapevine roots. The content of oxalate in the NaHCO_3_-treated roots was significantly enhanced from 3 to 24 h after treatment (HAT) and reached a maximum increase of 87.1% at 12 HAT compared to that in the control roots (Fig. 1a). The increased synthesis of oxalate promoted oxalate secretion and led to continuous accumulation in the culture solution; the oxalate content in the NaHCO_3_ culture solution reached 1.56 times that of the control solution at 24 HAT (Fig. 1g). Additionally, NaHCO_3_ treatment continuously significantly enhanced acetate synthesis but did not promote its secretion (Fig. 1f, l). In contrast, the other organic acids were generally not affected by NaHCO_3_ treatment. However, notably, the content of malate in the culture solution containing NaHCO_3_ was substantially increased at all time points (Fig. 1h). On the other hand, NaCl treatment as a positive control produced different effects on organic acid synthesis and secretion, e.g., NaCl imparted a reduced and stronger influence on oxalate and malate secretion, respectively, than NaHCO_3_ (Fig. 1g, h). Therefore, the changes in organic acid synthesis and secretion under NaHCO_3_ were attributed to both Na^+^ and ^–^HCO_3_.

### NaHCO_3_ treatment promotes H^+^ secretion of grapevine roots

**Fig. 2:**
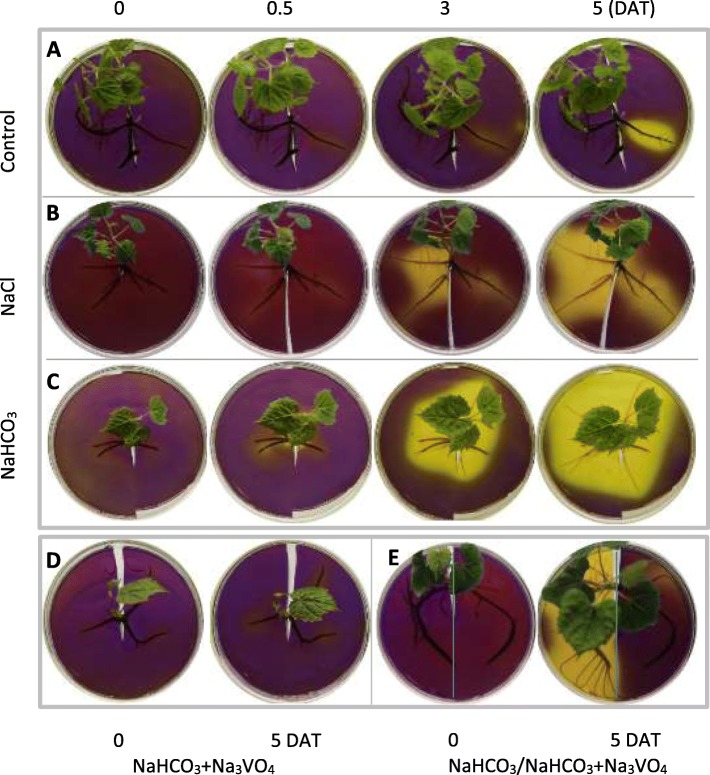
Effects of NaHCO_3_, NaCl and vanadate on rhizosphere acidification. The roots of five-week-old in vitro shoot cultures were treated with water (pH 7.0) (**a**), 75 mM NaCl (pH 7.0) (**b**), 75 mM NaHCO_3_ (pH 8.7) (**c**), and 75 mM NaHCO_3_ plus 0.1 mM Na_3_VO_3_ (**d**, **e**) for 6 h. After the roots were washed with deionized water, the roots were carefully spread on the bottom of a culture dish and then covered with an agar sheet containing 0.006% bromocresol purple and 0.8% agar. In addition to bromocresol purple and agar, Na_3_VO_4_ was added into the whole agar sheet in panel **d** and into the right side of the agar sheet in panel **e**

To detect root H^+^ secretion, the vine roots with different treatments were placed in solid medium with pH-sensitive bromocresol purple for 10 h (Fig. 2). Color changes occurred around the roots to indicate changes in pH. For the control roots, only less acidic substances were secreted, as indicated by the weak yellow color, at 0.5 and 3 days after treatment (DAT), and the acidic substances were gradually secreted and accumulated, generating a clear yellow color in the small area around the roots at 5 DAT (Fig. 2a). In contrast, the roots treated with NaHCO_3_ began to produce a clear yellow color at 0.5 DAT and produced large areas of yellow color at 3 and 5 DAT (Fig. 2c), indicating that large amounts of acidic substances had accumulated. Additionally, the NaCl treatment as a positive control led to a clear yellow color, but the area and intensity were smaller than those under NaHCO_3_ treatment (Fig. 2b, c); therefore, the production of acidic substances was attributed to both Na^+^ and ^−^HCO_3_. Moreover, the H^+^-ATPase inhibitor Na_3_VO_4_ almost completely inhibited the production of yellow color (Fig. 2d), suggesting that acid secretion is related to the PM H^+^-ATPase activity. Notably, the production of yellow color was accompanied by the occurrence of newly grown roots (Fig. 2a-c), and the production of yellow color and new roots was almost completely inhibited by the simultaneous treatment of NaHCO_3_ plus Na_3_VO_4_ (Fig. 2d). Further experimentation using solid medium, half of which contained Na_3_VO_4_, indicated that root growth and H^+^ secretion were substantially affected by H^+^-ATPase activity (Fig. 2e). Therefore, NaHCO_3_ treatment induced H^+^ secretion, which was accompanied by new root growth.

**Fig. 3:**
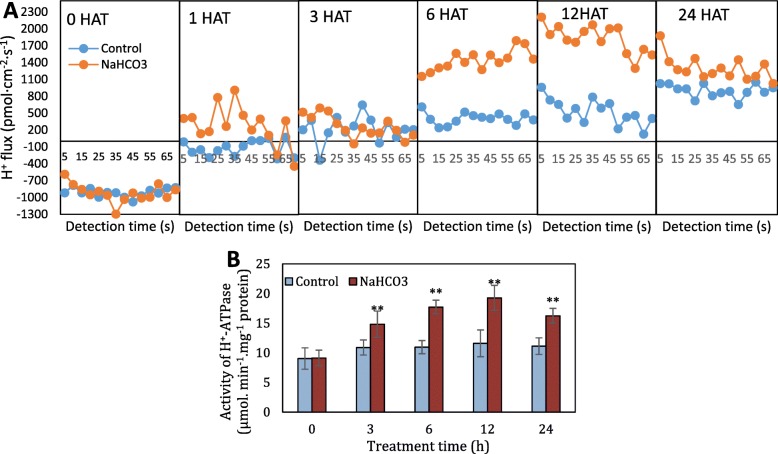
H^+^ flux (**a**) and H^+^-ATPase activity (**b**) of root tips in response to NaHCO_3_ treatment. Positive and negative values indicate the efflux and influx of H^+^, respectively. Values are the means of three replicates, and error bars denote the SD. ** highly significant difference, *P* < 0.01

On the other hand, H^+^ flux was determined using noninvasive microtest technology to further determine H^+^ secretion under NaHCO_3_ treatment (Fig. 3a). High negative values of H^+^ flux prior to treatment indicated the influx of H^+^ into root cells. In contrast, the H^+^ flux gradually changed to efflux, as indicated by the positive values in the control and NaHCO_3_-treated roots from 1 HAT. Compared to the low H^+^ efflux rate of the control roots, the H^+^ efflux rate of the roots treated with NaHCO_3_ was very high at 6 and 12 HAT. Additionally, the highest H^+^ efflux rate in the treated roots occurred at 12 HAT. On the other hand, the NaHCO_3_-treated roots possessed higher H^+^-ATPase activity than the control roots, and the highest activity of the treated roots was found at 12 HAT, which was 0.46-fold higher than that in the control (Fig. 3b).

### Identification of the changes in the transcriptome profile of grapevine roots in response to NaHCO_3_

**Fig. 4:**
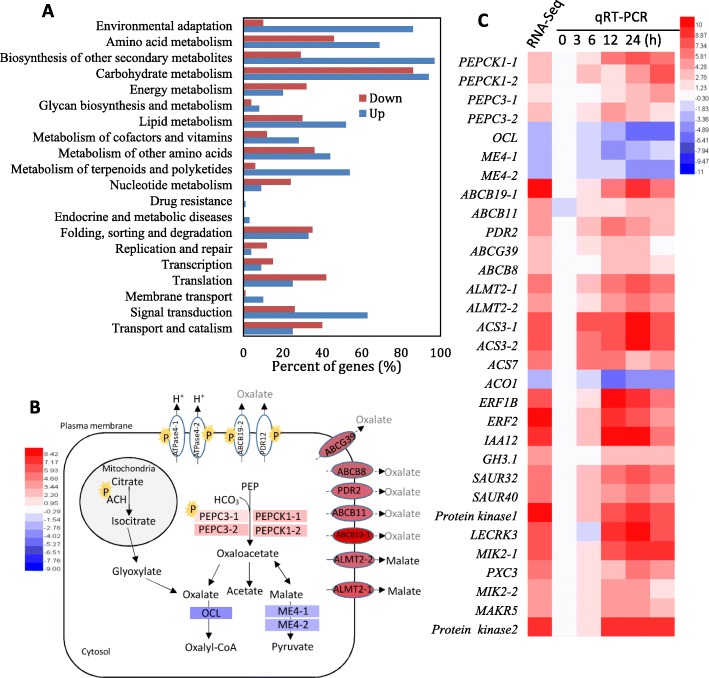
Functional categories (**a**) of DEGs responding to alkali stress and the key genes and proteins involved in the metabolism and extrusion of organic acid (**b**, **c**) and signaling related to ethylene, IAA and receptor kinases (**c**). RNA-Seq was performed using the top 10-mm length of grapevine roots after 12 h of NaHCO_3_ treatment. In panel **a**, functional categories of the DEGs from RNA-Seq were performed by Gene Ontology. In panel **b**, the schematic was constructed using the genes and proteins related to organic acid metabolism and extrusion. The normalized values of gene expression are shown on a color scale, and a P highlighted with a yellow background color denotes a phosphoprotein. The gray color indicates the possible transporters of oxalate. In panel **c**, the heatmap was constructed using the genes related to organic acid metabolism and transport, metabolism and signaling of ethylene and IAA, and receptor kinases. The normalized values of gene expression are shown on a color scale. Fold changes of qRT-PCR were calculated by comparing the relative expression values of the selected genes in the NaHCO_3_-treated and control vine roots

To explore the mechanism underlying the alkali stress-induced oxalate secretion, RNA-Seq analysis of the control and NaHCO_3_-treated vine roots was conducted to quantify gene changes. A total of 3232 and 1714 genes were up- and downregulated by at least one-fold in the NaHCO_3_-treated vine roots, respectively (Fig. 2), demonstrating that NaHCO_3_ treatment caused massive transcriptional reprogramming in the vine roots. All of the annotated differentially expressed genes (DEGs) were associated with 20 biological processes. The process of carbohydrate metabolism contained the most DEGs; additionally, biosynthesis of other secondary metabolites, environmental adaptation, amino acid metabolism and signal transduction were also clearly changed biological processes (Fig. 4a).

In the carbohydrate metabolism process, the gene expression of seven genes involved in oxalate and malate metabolism was significantly altered by NaHCO_3_ treatment (Fig. 2 and Fig. 4b). Two PEPCs and their kinases (PEPCKs) were significantly upregulated, positively contributing to the biosynthesis of oxaloacetate and thereby providing the substrate for oxalate and malate biosynthesis. An *oxalate--CoA ligase* gene (*OCL*) and two *NADP-malic enzyme* (ME) genes were significantly downregulated, which decreased the degradation of oxalate and malate, respectively. Additionally, the expression of five *ATP-binding cassette* (*ABC*) *transporters*, belonging to the process of membrane transport, was substantially increased, and in particular, the expression of *ATP-binding cassette B19* (*ABCB19*) increased 9.66-fold. Two *aluminum-activated malate transporters* (*ALMTs*) exhibited more than 5.0-fold higher expression in the NaHCO_3_-treated roots than in the control roots. *Plasma membrane* (*PM*) *H*^*+*^*-ATPases PMA2* and *AHA11* were only detected in the NaHCO_3_-treated roots but showed very low values of reads per kilobase of transcript per million mapped reads (RPKM), suggesting that they might not be the primary proton pump in grapevine roots.

With respect to the signal transduction process, the most significantly altered pathway was the ethylene signaling pathway, for which 51 genes related to ethylene biosynthesis and signaling were transcriptionally modified. Six *1-aminocyclopropane-1-carboxylic acid* (*ACC*) *synthases* (*ACSs*) were substantially upregulated by NaHCO_3_ treatment; in contrast, two *ACC oxidases* (*ACOs*) were transcriptionally changed, and *ACO1* was downregulated 2.97-fold (Additional file 1: Table S1). Additionally, IAA metabolism and signaling were also significantly changed. The expression of four genes related to IAA metabolism and signaling was upregulated more than 5-fold, including *indole-3-acetic acid-amido synthetase* (*GH3*) and three *IAA responsive factors* (*IAA12*, *SAUR32* and *SAUR40*). Moreover, six protein kinases located in the plasma membrane were substantially upregulated by NaHCO_3_ treatment, suggesting that plasma membrane proteins were regulated by phosphorylation.

On the other hand, 34 differentially expressed genes, which contained genes involved in organic metabolism and transport and hormone biosynthesis and signaling, were detected by qRT-PCR at different times after NaHCO_3_ treatment. Similar expression changes in the DEG tag profiles were found, which not only validated the reliability of the DEGs but also demonstrated their expression patterns under alkali stress (Fig. 4c).

### Quantitative analysis of phosphoproteins with phosphorylation levels significantly changed in response to NaHCO_3_

We identified 2669 unique phosphoproteins, collectively containing 6312 nonredundant phosphorylation sites. Among those phosphorylation sites, 5404 (85.6%) were found at serine, 877 (13.9%) were found at threonine and 31 (0.49%) were found at tyrosine residues. Of the 2669 phosphoproteins, 2141 phosphoproteins were identified, which contained different quantities of phosphorylation sites (from 1 to 25); a total of 1822 and 608 phosphoproteins contained one and two phosphorylation sites, respectively (Additional file 2: Table S2).

**Fig. 5:**
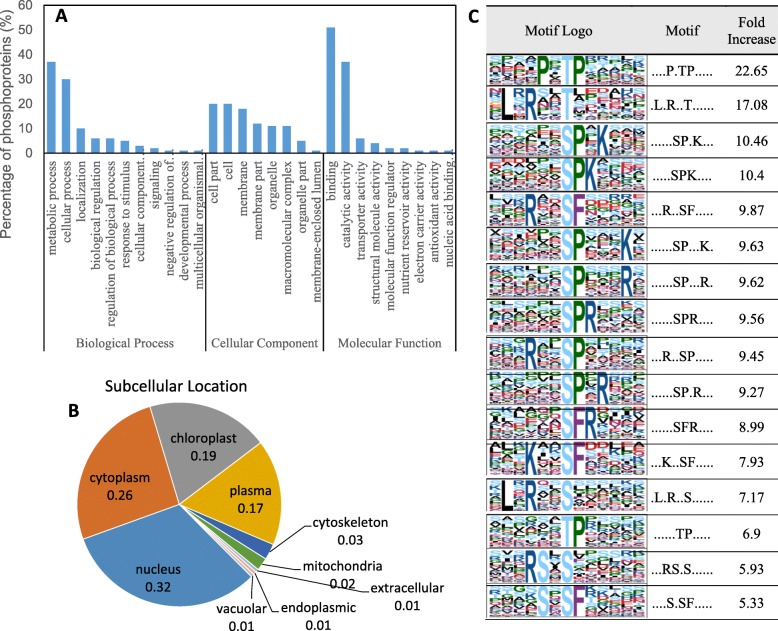
The distribution of differentially phosphorylated proteins (**a**, **b**) and significantly enriched phosphorylated sites (**c**). The 197 phosphorylated proteins were classified according to their biological process, cellular component, molecular function and subcellular location (**a**, **b**). The Gene Ontology (GO) annotation proteome was derived from the UniProt-GOA database (www. http://www.ebi.ac.uk/GOA/). Soft motif-x was used to analyze the model of sequences constituted with amino acids in specific positions of modify-21-mers (10 amino acids upstream and downstream of the site, but phosphorylation with modified-13-mers was 6 amino acids upstream and downstream of the site) in all protein sequences. All of the database protein sequences were used as background database parameters and other parameters with default

When comparing the phosphorylation levels between the NaHCO_3_-treated and control samples, a total of 197 phosphoproteins (270 phosphorylation sites) showed a significant change (ratio ≥ 1.5, *P* < 0.05), with 107 upregulated and 163 downregulated (Additional file 2: Table S2). The 197 phosphoproteins were annotated using Blast2GO according to the biological process, cellular component and molecular function (Fig. 5a). The phosphoproteins were classified into 11 biological processes, with metabolic process, cellular process and localization as the top three categories. For the molecular function, phosphoproteins were classified into 9 categories, and the top 3 categories were binding, catalytic activity and transporter activity. For the cellular components, cell part, cell and membrane possessed the highest number of phosphoproteins (Fig. 5a). The phosphoproteins modified by NaHCO_3_ were primarily localized in the nucleus, cytoplasm, chloroplast and plasma (Fig. 5b). Additionally, Motif-X analysis identified 16 significantly enriched motifs (Fig. 5c).

Aconitate hydratase (ACH) and PEPC3–1, which are involved in oxalate and malate synthesis, were significantly phosphorylated in the NaHCO_3_ treatment compared to the control (Fig. 4b; Table 1). The phosphorylation levels of two plasma membrane-localized ATPases (ATPase4–1 and particularly ATPase4–2) were substantially enhanced, which are responsible for H^+^ efflux and provide energy for the transport of oxalate and malate across the plasma membrane. Additionally, two plasma membrane ABC transporters ABCB19–2 and pleiotropic drug resistance 12 (PDR12), potential transporters of oxalate, were also significantly phosphorylated. Moreover, the phosphorylation levels of two plasma membrane-localized serine/threonine-protein kinases (STY46 isoform X1 and CDL1), ACO4 and ACO11 for ethylene synthesis and EIN2 for ethylene signaling were significantly changed (Fig. 4b; Table 1).

**Table 1:** Phosphorylation levels of the proteins involved in organic acid metabolism and transport and signaling in grapevine roots regulated by NaHCO_3_ stress

Protein accession	Modified sequence (Probabilities)	Position	NaHCO_3_/CK Ratio	*P* value	Protein description	Gene name	Subcellular Localization
D7T1R6	IIDWENS(0.244)S(0.756)PK	92	2.32	0.000522	ACH	VIT_00s0264g00030	mitochondria
F6H2N7	MAS(1)IDAQLR	11	1.74	0.0414	PEPC3–1	VIT_19s0014g01390	cytoplasm
D7SQD1	T(1)LHGLQPPETSNLFNDK	886	3.46	0.000023	ATPase 4–1	VIT_11s0052g00620	PM
D7SQD1	GLDIDTIQQHYT(1)V	953	2.43	0.00746	ATPase 4–1	VIT_11s0052g00620	PM
D7SQD1	GHVES(1)VVK	936	2.10	0.00126	ATPase 4–1	VIT_11s0052g00620	PM
D7SQD1	ELS(1)EIAEQAK	909	1.59	0.00464	ATPase 4–1	VIT_11s0052g00620	PM
F6H3A8	S(1)IGLEEIK	6	1.97	0.00178	ATPase 4–2	VIT_04s0008g02460	PM
F6H3A8	GHMES(1)VVK	936	1.94	0.000255	ATPase 4–2	VIT_04s0008g02460	PM
F6H3A8	T(1)LHGLQPPETSNIFSDK	886	1.62	0.00172	ATPase 4–2	VIT_04s0008g02460	PM
F6H3A8	GLDIDTIQHHYT(1)V	953	1.51	0.00762	ATPase 4–2	VIT_04s0008g02460	PM
F6H5R3	NLSYQY(0.002)S(0.953)T(0.045)GADGR	645	1.94	0.0407	ABCB19–2	VIT_14s0108g00430	PM
F6H5R3	LSHS(0.03)LS(0.964)T(0.006)K	626	1.85	0.004	ABCB19–2	VIT_14s0108g00430	PM
F6HX69	S(0.986)S(0.014)GADVFSR	22	1.86	0.0194	PDR 12	VIT_09s0002g05600	PM
F6HX69	S(0.933)S(0.067)RDEDDEEALK	31	1.56	0.000376	PDR12	VIT_09s0002g05600	PM
F6HFE2	GLVDS(0.996)GITTIPR	65	0.61	0.0059	ACO 4	VIT_01s0011g05650	chloroplast
F6HKN0	GLSDS(0.997)GIT(0.002)SIPR	28	0.59	0.0015	ACO 11	VIT_08s0007g03040	cytoskeleton
F6HR32	EISGSSPSLTSEGPGS(1)FR	648	1.66	0.0201	EIN 2	VIT_08s0040g01730	PM
D7U4Z3	QSWPNHHS(0.014)LS(0.976)PT(0.011)GEQEETGIK	266	1.55	0.0311	STY46 isoform X1	VIT_03s0038g03040	cytoplasm
F6HDG9	DGSSAQS(1)HHVTR	36	3.535	0.00204	CDL1	VIT_05s0020g01770	cytoplasm
F6HDG9	LGS(0.989)PS(0.01)T(0.001)HKNS(1)PDFR	420	1.955	0.0332	CDL1	VIT_05s0020g01770	cytoplasm
F6HDG9	LGS(0.989)PS(0.01)T(0.001)HKNS(1)PDFR	413	1.955	0.0332	CDL1	VIT_05s0020g01770	cytoplasm

*PM* Plasma membrane, *ACH* Aconitate hydratase, *PEPC* Phosphoenolpyruvate carboxylase, *CDL* Serine/threonine-protein kinase CDL, *ACO* 1-aminocyclopropane-1-carboxylate oxidase, *EIN2* Ethylene-insensitive protein 2. The values of the NaHCO_3_/CK ratio are presented as the means of three replicates

### Organic acid secretion by vine roots involves ethylene and IAA

**Fig. 6:**
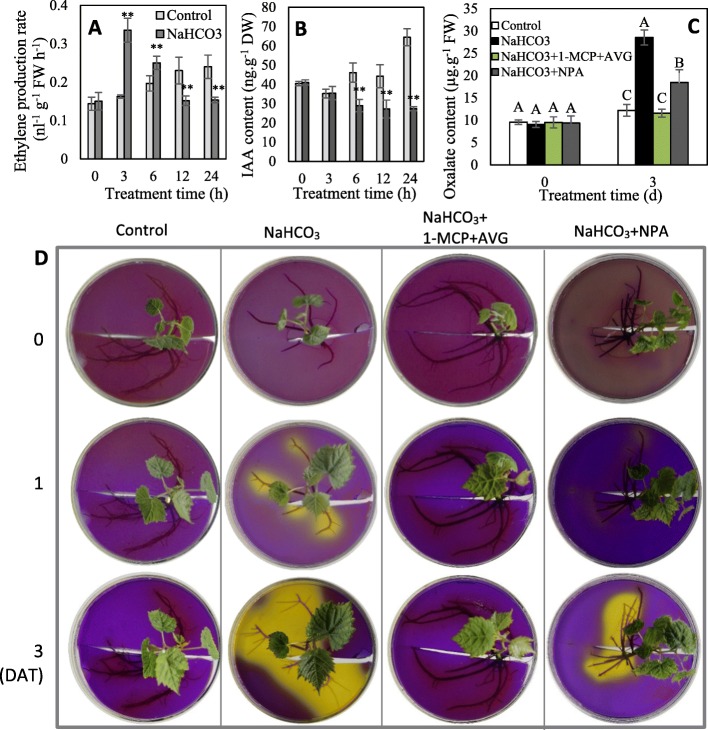
Changes in ethylene (**a**) and IAA (**b**) and the effects of their inhibition on organic acid secretion (**c**, **d**). 1-MCP, 1-methylcyclopropene, an inhibitor of ethylene perception; AVG, 1-N-naphthylphthalamic acid, an inhibitor of IAA transport; and AVG, aminoethoxyvinylglycine, an inhibitor of ethylene biosynthesis. DAT, days after treatment. In panels **a**-**c**, values represent the means ± SD of three replicates. ** highly significant difference, *P* < 0.01; values indicated by different capital letters are significant at *P* < 0.01 at the same treatment time

Transcriptomic and/or phosphoproteomic profiling indicated that the metabolism and signaling of IAA and ethylene were significantly altered by NaHCO_3_ treatment. To reveal the role of ethylene and IAA in mediating NaHCO_3_-induced organic acid secretion, the changes in ethylene and IAA under NaHCO_3_ treatment and the effects of the inhibition of ethylene and IAA biosynthesis and/or signaling on organic acid secretion were determined. Compared with the control, NaHCO_3_ treatment significantly increased ethylene production at 3 and 6 HAT but substantially decreased ethylene production at 12 and 24 DAT. In contrast, the IAA content was substantially reduced by NaHCO_3_ at 6 DAT, and the decrease in the IAA content reached 57.6% at 24 HAT (Fig. 6a, b). Additionally, the inhibition of ethylene biosynthesis and perception via aminoethoxyvinylglycine (AVG) and 1-methylcyclopropene (1-MCP) completely blocked NaHCO_3_-induced organic acid secretion, and the inhibition of IAA biosynthesis via 1-N-naphthylphthalamic acid (NPA) significantly reduced NaHCO_3_-induced organic acid secretion (Fig. 6c, d). Therefore, ethylene signaling is necessary to regulate organic acid secretion under NaHCO_3_, and IAA also participates in the process; additionally, their roles are largely affected by their concentrations in plants.

## Discussion

### Accumulation and secretion of oxalate are important physiological responses of grapevine roots to alkali stress

Plant roots are in direct contact with the soil and can adjust to an adverse rhizosphere environment by secreting a large amount of compounds, which is the most direct and obvious response of roots to environmental stresses [5]. Under alkali stress, the accumulation of organic acids is observed in many plants. Oxalate was highly accumulated and secreted by grapevine roots under alkali stress (Fig. 1a, g). A large amount of oxalate was also found in the roots of *Kochia sieversiana* and *Suaeda glauca* under alkali stress [22, 23]. In contrast, *Chloris virgata* was found to predominantly accumulate citrate, and wheat roots secrete large amounts of lactic, acetic and formic acids under alkali stress [24, 25]. The accumulation and secretion of organic acids, including oxalate, citrate and malate, play important roles in osmoregulation, pH adjustment, and ionic balance maintenance by providing a negative charge under alkali and salt stresses [5, 22, 23]. This study demonstrated that both NaHCO_3_ and NaCl stimulated the secretion of organic acids, but NaHCO_3_ created a more acidic rhizosphere environment (Fig. 2b, c). Additionally, NaHCO_3_ and NaCl imparted different effects on oxalate, acetate and malate (Fig. 1). Similarly, previous reports have also demonstrated that alkali and salt stress have different effects on organic acid metabolism and secretion. For example, the concentration of citrate, the most dominant organic acid, is substantially increased by alkali stress but decreased by salt stress in *Puccinellia tenuiflora* roots; additionally, alkali stress causes the secretion of citric acid into the rhizosphere, while salt stress does not [5]. Different changes in citrate and malate were also found in *Chloris virgata* under alkali and salt stresses [25].

Therefore, organic acid secretion is a widespread physiological response of plants, but oxalate is the primary organic acid synthesized and secreted by vine roots. Additionally, ^−^HCO_3_ plays a key role in inducing oxalate synthesis and secretion in grapevine roots.

### The possible pathway of organic acid synthesis and secretion based on the key genes and phosphoproteins under alkali stress

Three pathways for oxalate biosynthesis have been proposed in plants, i.e., the glycolate/glyoxylate pathway, the ascorbate pathway and the oxaloacetate (OAA) pathway [26, 27]. Under alkali stress, substantial amounts of ^−^HCO_3_ enter the root and provide the substrate for PEPCs, which catalyze the β-carboxylation of PEP to OAA using ^−^HCO_3_ in the cytosol in an irreversible process [28]; thereafter, OAA can be converted to oxalate, acetate and malate (Fig. 4b). In *Arabidopsis*, the *pepc3* mutant abolished the salt stress-induced increase in malate, suggesting the role of *AtPEPC3* in regulating organic acid synthesis [29]. The significant upregulation of the expression of two *PEPC3* genes under alkali stress might enhance OAA synthesis and the subsequent conversion of OAA to oxalate and acetate (Fig. 4b, c). In addition to transcriptional regulation, PEPC activity is also regulated by phosphorylation catalyzed by phosphoenolpyruvate carboxylase kinase (PEPCK) [20]. PEPC phosphorylation is abolished in the *pepc3* mutant of *Arabidopsis* under salt stress [20], suggesting that PEPC3 might be the target protein of PEPCKs. The significant increases in the expression levels of two *PEPCK1* genes and the phosphorylation level of PEPC3–1 also suggested the phosphorylation regulation of PEPC3–1 by PEPCK1s (Fig. 4b, c). Collectively, the above results suggest that the OAA pathway is the main pathway for oxalate and acetate biosynthesis under alkali stress. However, notably, the glycolate/glyoxylate pathway might also be modified in grapevine roots under alkali stress, as indicated by the significant increase in the phosphorylation level of aconitate hydratase (aconitase), a key protein in the glycolate/glyoxylate pathway [30].

H^+^ extrusion and the transport of organic acid anions across the plasma membrane are controlled by PM H^+^-ATPases, providing energy by creating an electrochemical proton gradient for transporters [31]. The PM H^+^-ATPase is important for the root proton-secretion adaptation to alkaline stress [15]; high PM H^+^-ATPase activity and proton secretion have been shown to enhance plant tolerance to alkaline stress [32], while lower PM H^+^-ATPase activity and proton secretion result in sensitity to alkaline stress [33]. PM H^+^-ATPase activity is tightly regulated by phosphorylation at several N- and C-terminal residues, especially upon exposure to various environmental stimuli [34]. The significant increase in the phosphorylation levels showed that the two ATPases 4 are the key proton pumps in grapevine roots under alkali stress (Fig. 4b, Table 1). The ATPases 4 from *Arabidopsis* (AHA4) and tobacco (PMA4) plants are also reported to be involved in salt tolerance [35, 36]. Therefore, ATPase 4 might function in plant tolerance to alkali and salt stress.

In contrast, the specific transporter of oxalate remains unclear. Nevertheless, some experiments have indicated the role of ABC transporters in transporting organic acids in plants. Four ABC transporters (ABCG11, ABCG21, ABCA2, and ABCB21) were considered to be potentially involved in oxalate and/or citrate secretion under Al stress in *Grain amaranth* roots [10]. AtPDR6, belonging to the ABCG subfamily of the ABC transporter family, is involved in the root extrusion of organic acids, including succinate, fumarate and malate [37]. In this study, the expression levels of five *ABC transporter*s, particularly *ABCB19–1*, were substantially increased by NaHCO_3_ treatment (Fig. 4b, Additional file 1: Table S1), suggesting their association with organic acid secretion. In particular, the expression of *ABCB19–1* was well correlated with the oxalate content in roots (Fig. 4c, Fig. 1a), suggesting its role in transporting oxalate. Additionally, two ABC transporters, ABCB19–2 and PDR12, were significantly phosphorylated (Table 1), and PDR12 has been reported to mediate the extrusion of water-soluble carboxylate anions in yeast [38]; therefore, ABCB19–2 and PDR12 might participate in the transport of organic acids. However, the detailed function of the above transporters must be characterized in future studies.

ALMTs are found throughout plant genomes and are involved in a range of distinct functions in different cell types. ALMT1 functions in mediating organic acid secretion in the Al-tolerance response in many plant species [39]. In contrast, the ALMT2 transporter mediates an Al-independent electrogenic transport of organic anions, such as malate and citrate, across the plasma membrane in wheat [40]. The upregulation of two *ALMT2s* (Fig. 4b, c) and the increased content of malate implied that ALMT2 transporters mediate the salt- and alkali-induced malate extrusion in vine roots. Notably, a large increase in malate secretion was not accompanied by its accumulation under salt and alkali stress (Fig. 1b, h). A similar phenomenon was also found in other plants under Al stress [22, 41]. Therefore, it is suggested that malate metabolism is not a limiting factor but rather that transporters are more important for its secretion under alkali stress.

### The signaling pathway mediating alkali stress-induced organic acid secretion

Ethylene is an important signaling molecule mediating numerous important biological processes, including responses to abiotic stresses [42]. In this study, the application of inhibitors related to ethylene biosynthesis and perception indicated the necessity of ethylene in organic acid secretion under alkaline stress (Fig. 6c, d). However, the exogenous application or endogenous overproduction of ethylene substantially inhibited H^+^-ATPase activity and H^+^ efflux in rice roots under alkali stress [17]; additionally, ethylene production was reduced with NaHCO_3_ treatment (Fig. 6a). Similarly, transgenic tobacco plants with poor ethylene biosynthesis exhibited elevated salt tolerance, while rice plants treated with ethylene exhibited salt hypersensitivity [43, 44]. Therefore, the role of ethylene might be concentration-dependent [42, 45], and low-concentration ethylene might be necessary to mediate NaHCO_3_-induced organic acid secretion. Ethylene biosynthesis is mainly regulated by ACS and ACO at the transcriptional and posttranslational levels [46, 47]. When grapevines were subjected to alkali stress, the decline in ethylene production was accompanied (Fig. 6a) by the decreased expression of *ACO1* (Fig. 4c) and phosphorylation levels of ACO4 and ACO11 (Table 1) but the increased expression of *ACS3* and *ACS7* (Fig. 4c), suggesting the key role of ACOs in regulating ethylene synthesis under alkali stress. Additionally, EIN2, a central regulator of ethylene signaling [48], controls the transduction of the ethylene signal from the ER membrane to the nucleus in *Arabidopsis* [48], and its phosphorylation inhibited ethylene signaling (Table 1; 48). After the signal cascade mediated by EIN5, EIN6, EIN3 and others, ethylene signals are delivered to ethylene responsive factors (ERFs), the last downstream components of the ethylene signaling pathway, which lead to the regulation of ethylene controlled gene expression [43]. Here, the changes in the expression of a large amount of ERFs may correspond to the different biological processes regulated by ethylene under alkali stress.

Auxin has been reported to control root apoplastic acidification, to enhance the Al-induced exudation of citrate and to promote the phosphorylation of PM H^+^-ATPases [13, 45, 49]. Additionally, PIN2 (an auxin efflux transporter) activates plasma membrane H^+^-ATPases to release protons, which is necessary for the adaptation of *Arabidopsis* to alkali stress [15]. Therefore, auxin plays a key role in regulating organic acid secretion under alkali stress. A recent study found that endogenous auxin controls apoplastic acidification; however, an endogenous increase in the overexpression of auxin biosynthesis gene or exogenous increase in the auxin level induces a transient alkalinization [45], which is similar to the role of ethylene discussed above. Here, NaHCO_3_ treatment induced a decrease in IAA, but NPA treatment reduced organic acid secretion (Fig. 6b-d), suggesting that the fine-tuning of IAA biosynthesis may be essential for the regulation of organic acid secretion. Additionally, the genes of *IAA12* and *GH3* as IAA repressors [45, 50] were also substantially upregulated (Fig. 4c), probably reducing IAA synthesis and signaling. Small auxin-up RNA (SAUR) genes represent the largest family of auxin-responsive genes and participate in auxin-mediated PM H^+^-ATPase activation [45, 51]. The large increase in the expression of *SAUR32* and *SAUR40* suggests that they likely regulate organic acid secretion in response to auxin in vine roots. Particularly, it has been demonstrated that auxin mediates ethylene signaling to control root growth [52]. Therefore, we inferred that ethylene most likely regulates organic acid secretion through auxin signaling in vine roots under alkali stress.

On the other hand, the cell membrane harbors hundreds of different receptor kinases that receive environmental signals at the receptor domain on the extracellular side of the membrane and convert these signals into cellular responses via an intracellular protein kinase domain [34]. The PM H^+^-ATPase was reported to interact with multiple such receptor kinases [53]. The receptor kinase-mediated control of primary active proton pumping at the plasma membrane, e.g., PSY1R, increases proton efflux from roots by interacting with and phosphorylating AHA2/AHA1 [54]. In this study, the high-level expression and/or phosphorylation of the 11 plasma membrane-located receptor kinases might participate in the regulation of ATPases. Although the exact roles of candidate genes remain to be examined, our data provide a platform for further functional analyses of these genes.

## Conclusion

Oxalate was the primary organic acid synthesized and secreted by vine roots under NaHCO_3_ stress. The OAA pathway, including two PEPC3s and PEPCK1s, plays a key role in oxalate synthesis. The secretion of organic acids and H^+^ were controlled by PM H^+^-ATPases, and two phosphorylated PM ATPases 4 were the main proton pumps under NaHCO_3_ stress. Additionally, ABCB19–2 and PDR12 might participate in the transport of oxalate and other organic acids. Low-concentration ethylene mediates NaHCO_3_-induced organic acid secretion, and IAA also participates in this process.

## Methods

### Determination of organic acids in grapevine roots and root exudate solutions

Healthy apical growth tips of A15 vines were removed in early summer to establish grapevine in vitro shoot cultures. The shoot cultures were subcultured on Murashige and Skoog medium containing 3% (w/v) sucrose, 7 g. L^− 1^ agar and 0.2 mg. L^− 1^ IBA. Five-week-old grapevine in vitro shoot cultures were transferred to glass bottles with a 10-cm height and 5-cm diameter. The vines were treated with 50 ml water (pH 7.0) as a control, 75 mM NaCl (pH 7.0) and NaHCO_3_ (pH 8.7). Each glass bottle was provided sufficient oxygen with an oxygen machine (SenSen Group, China). The vines were grown in a controlled-environment growth cabinet at 25 °C, a 16-h photoperiod and a light intensity of 600 μmol/m^2^/s. At different time points after different treatments, the roots were collected and immediately frozen in liquid nitrogen for organic acid extraction. The treatment solution was collected and evaporated to dryness with a rotary evaporator, and the residue was dissolved in 1 ml double distilled water. The filtrate, which was passed through a 0.45-μm filter, was used for organic acid determination. The extraction of organic acids from the roots and their determination for root extract and treatment solutions were performed using a capillary electrophoresis system (Beckman P/ACE, Palo Alto, CA) as described in our previous study [55].

### H^+^ secretion test

H^+^ secretion was detected according to a previously described method [56]. Five-week-old grapevine A15 in vitro shoot cultures were treated with water (pH 7.0), 75 mM NaCl (pH 7.0), 75 mM NaHCO_3_ (pH 8.7), and 75 mM NaHCO_3_ plus 0.1 mM Na_3_VO_3_ for 6 h. Then, the roots were rinsed, carefully spread in Petri dishes and covered by solid medium (pH 5.8) which consisted of 0.006% bromocresol purple (pH indicator, discoloration range of 5.2–6.8), 1 mmol l^− 1^ CaSO_4_, 2.5 mmol l^− 1^ K_2_SO_4_ and 0.8% agar. The vines were grown in a controlled-environment growth cabinet at 25 °C and continuous illumination at 400 μmol/m^2^/s light intensity.

### Measurement of H^+^-ATPase activity and H^+^ flux in roots

Plant materials were the same as those described in the organic acid determination Section. The extraction of plasma membrane (PM) protein and activity determinations of PM H^+^-ATPase of the root tips were conducted according to the method of Yan et al. (2002) [56]. The activity of H^+^-ATPase was determined by the Pi amount after 30 min of reaction in 0.5 ml of 30 mM BTP/MES buffer (pH 6.5), 5 mM MgSO_4_, 50 mM KCl, 1 mM Na_2_MoO_4_, 1 mM NaN_3_, 5 mM ATP, and Brij 58 (0.02% w/v). The reaction was initiated by adding 5 mg of membrane protein and stopped with 1 ml of stopping reagent (2% H_2_SO_4_, 5% SDS, and 0.7% (NH_4_)_2_MoO_4_) followed immediately by the addition of 50 μl ascorbic acid (10% w/v). The color development was completed after 30 min. The absorbance at 820 nm was measured using a spectrophotometer.

Root H^+^ fluxes were measured using the scanning ion-selective electrode technique with a Noninvasive Microtest Technology System (NMT, Xuyue Beijing Sci. & Tech. Co., Ltd., Beijing, China). The 10-mm long roots from the root apex were equilibrated in the measurement solution for 20 min and then immobilized in the measurement chamber in 5 ml of fresh measuring solution.

### RNA-Seq and quantitative real-time PCR (qRT-PCR)

Total RNA of 10-mm-long grapevine roots from the apex was extracted using TRIzol Reagent (Invitrogen, Carlsbad, CA, USA), and mRNA was isolated using poly-T oligo attached magnetic beads. Transcriptome sequencing and analysis were conducted by OE Biotech Co., Ltd. (Shanghai, China). Sequencing libraries, constructed using NEBNext® Ultra™ RNA Library Prep Kit for Illumina® (7530 L, NEB, United States), were sequenced on an Illumina HiSeq 4000 platform, and 150-bp paired-end reads were generated. Clean reads were assembled into transcripts using Cufflinks referencing the grape genome (http://genomes.cribi.unipd.it/grape/). The unigene expression levels were quantified using RPKM. Unigenes that were differentially expressed between two samples were screened using a false discovery rate < 0.05 and absolute log_2_ (fold change) ≥1 as the threshold. Three biological replicates were generated for the control and NaHCO_3_ treatment.

qRT-PCR was performed using SYBR Green MasterMix (SYBR Premix EX Taq TM, Dalian, China) on a Bio-Rad iQ5 (Hercules, CA, United States) instrument, and the primers are listed in Additional file 3: Table S3.

### Phosphopeptide and phosphoprotein identification and quantification

Proteins from grapevines were extracted and quantified with the BCA kit (Beyotime, Beijing, China). After trypsin digestion, the peptide mixture was desalted on a Strata X C_18_ SPE column (Phenomenex, Torrance, CA, USA). Each peptide was vacuum-dried and reconstituted in 0.5 M TEAB (Sigma, USA). The peptide mixture was labeled using TMT kit (Thermo Fisher Scientific, Torrance, CA, USA) according to the manufacturer’s introduction. The TMT labeled peptides were fractionated by high pH reverse-phase HPLC and concentrated by vacuum centrifugation. The phosphopeptides were enriched using IMAC microspheres, and they were then eluted with elution buffer, followed by lyophilization and LC-MS/MS analysis. The phosphopeptides were dissolved in 0.1% formic acid (solvent A) and loaded onto a C_18_-reversed phase column (15-cm length, 75 μm i.d., packed in-house) and separated with a linear gradient of solvent B (0.1% formic acid in 98% acetonitrile) at a constant flow rate of 0.4 μL/min on an EASY-nLC 1000 UPLC system (Thermo). The peptides were eluted with a gradient of 6 to 23% solvent B for 26 min, 23 to 35% solvent B for 8 min, and 80% solvent B for 6 min. The peptides were detected and identified by tandem mass spectrometry (MS/MS) in Q ExactiveTM Plus (Thermo) coupled online to the UPLC, which was supported by Jingjie PTM BioLabs (Hangzhou, China). MS/MS data were searched using a Maxquant search engine (v1.5.2.8) against the *Vitis vinifera* proteome concatenated with reverse decoy database. The parameters in Maxquant searches were as follows: max missing cleavage of Trypsin/P, 2; peptide mass tolerance, 20 ppm in the first search and 5 ppm in the main search; MS/MS tolerance, 0.02 Da; fixed modification, carbamidomethyl on Cys; variable modification, oxidations on Met. FDR was adjusted to < 1%.

### IAA extraction and determination

IAA extractions were performed according to our previous study [57]. Separation and quantification of IAA were carried out using a Scientific Ultimate 3000 HPLC system (Thermo, San Jose, CA, USA) coupled to a TSQ Quantum Access MAX system (Thermo, San Jose, CA, USA). HPLC separation was performed using a Thermo Scientific Hypersil Gold column (50 × 2.1 mm, 1.9 μm). The injection volume was 10 μL. The mobile phase consisted of 0.5% acetic acid in water (A) and methanol (B) with the following gradient at a flow rate of 1.0 ml.min^− 1^: 0–0.5 min, 0–20% B; 0.5–3.0 min, 20–90% B; 3.0–6.5 min, 90% B; 6.5–10.0 min, 90–20% B; 10.0–15.0 min, 20% B. Detection and quantification of IAA were performed using the ESI negative mode. The parameters were set as follows: parent mass by charge (m/z) of 263.1, daughter mass by charge (m/z) of 153.0, and a collision energy of 14 eV.

### Determination of the ethylene production rate

The ethylene production rate was measured using a GC-9A gas chromatograph (Shimadzu, Japan) equipped with a GDX-502 column and a flame ionization detector. The vine roots were enclosed in a 5-mL centrifuge tube with sealing film and incubated at 25 °C for 3 h. Five milliliters of the headspace gas was withdrawn from each tube through the septum stopper using a gas-tight syringe and assayed.

### Statistical analyses

Statistical analysis was performed with the SPSS (V19.0) statistical software package. A one-way analysis of variance followed by Duncan’s multiple range test was employed.

## Additional files

Additional file 1: **Table S1.** RNA-Seq profiles of the control and NaHCO_3_-treated vine roots. Unigenes differentially expressed between two samples were screened using a false discovery rate < 0.05 and absolute log_2_ (fold changes) ≥ 1 as the threshold. (XLSX 1337 kb)

Additional file 2: **Table S2.** Analysis of phosphopeptide changes in grapevine roots exposed to NaHCO_3_ stress. The significantly altered phosphoproteins between two samples were screened using fold changes ≥1.5 or ≤ 0.67 (*P* < 0.05). (XLSX 96 kb)

Additional file 3: **Table S3.** Primer sequences for real-time quantitative RT-PCR. Gene ID is derived from the grape genome (http://genomes.cribi.unipd.it/grape/). (DOCX 17 kb)

## Data Availability

The mass spectrometry proteomics data have been deposited to the ProteomeXchange Consortium via the PRIDE partner repository with the dataset identifier PXD013746 (http://www.ebi.ac.uk/pride). Full RNA-Seq data were submitted to the sequence read archive (SRA) of NCBI under BioSample accessions SAMN11579694 and SAMN11579695 (https://www.ncbi.nlm.nih.gov/sra).

## Abbreviations

1-MCP: 1-methylcyclopropene
ABCB19: ATP-binding cassette B19
ACC: 1-aminocyclopropane-1-carboxylic acid
ACH: Aconitate hydratase
ACO: 1-aminocyclopropane-1-carboxylic acid oxidase
ACS: 1-aminocyclopropane-1-carboxylic acid synthase
ALMT: Aluminum-activated malate transporter
AVG: Aminoethoxyvinylglycine
DAT: Days after treatment
DEG: Differentially expressed gene
HAT: Hours after treatment
IAA: Indoleacetic acid
ME: Malic enzyme
NPA: 1-N-naphthylphthalamic acid
OCL: Oxalate-CoA ligase
PDR12: Pleiotropic drug resistance 12
PEPC: Phosphoenolpyruvate carboxylase
PEPCK: PEPC kinase
PM: Plasma membrane
qRT-PCR: quantitative real-time PCR
RPKM: Reads per kilobase of transcript per million mapped reads
