# Supplementary material for: Transcriptomic and phosphoproteomic profiling and metabolite analyses reveal the mechanism of NaHCO3-induced organic acid secretion in grapevine roots
Source: BMC Plant Biol. 2019 Sep 3;19:383. doi: 10.1186/s12870-019-1990-9 (PMC6724372; doi:10.1186/s12870-019-1990-9)
Supplement: Supplementary file 3 — Table S3. Primer sequences for real-time quantitative RT-PCR. Gene ID is derived from the grape genome (http://genomes.cribi.unipd.it/grape/). (DOCX 17 kb) [file 12870_2019_1990_MOESM3_ESM.docx]

| **Gene_id** | **Gene_symbol** | **Forward primer (5'-3')** | **Reverse primer (5'-3')** |
| --- | --- | --- | --- |
| VIT_214s0036g00420 | *PEPCK1-1* | ATTACCAACTGTGCGAGGAG | GAAGAATGTGAGGGTGAGGA |
| VIT_205s0049g00950 | *PEPCK1-2* | GATGACAAGCGTTGGTGAGAC | CTACAGGGAAGGCATAGGGGT |
| VIT_212s0028g02180 | *PEPC3-1* | ATTTCACCATGTCCCTTCAG | TTAGATACTTGTTCAGAGTCGTG |
| VIT_219s0014g01390 | *PEPC3-2* | GTGATCCTTGTGACTTGAATG | TCAACCAGTTCTTCCATTAAC |
| VIT_202s0025g03660 | *OCL* | GTAGCATGGGAAGGCTCTG | TTGATGGCAAATGTAGCTCC |
| VIT_209s0002g03620 | *ME4-1* | TGGGTCATAGTCTTCAACAATC | CGTACAGAACCAGGCTCCTAAC |
| VIT_204s0008g00180 | *ME4-2* | AGTACTCCAAGTTCTAGCATGC | CTAAAGACCTCCAAGCATCC |
| VIT_216s0098g00570 | *ABCB19-1* | TCTGTCACATGCGTTGGC | CAGTTAGGCAATATTATGGCAC |
| VIT_205s0020g00890 | *ABCB11* | TATGAGTGCTTCATCTTAGTGAG | GACTTTAGTATTCCATTGGGATG |
| VIT_206s0061g01490 | *PDR2* | GGTTAGGACATAGGGTGTAGA | CATTTATGGTAAATCGACAGG |
| VIT_204s0008g04820 | *ABCG39* | TGTTTCATGAATCCTAGAGATG | CACTAAATTGGCTTCATTGTC |
| VIT_211s0016g03160 | *ABCB8* | TGTTGAGCTCTGAAAGATGG | TCAGAGGAACCAACAAGTTC |
| VIT_206s0009g00450 | *ALMT2-1* | ATTCAAGCTTAGGCATGTGAG | TATATGGCTGACAGAAGAACAT |
| VIT_206s0009g00480 | *ALMT2-2* | GTAAACAGCTGCTGCATTGTAG | AGACTCTTCCGCTAATGTCTC |
| VIT_200s0840g00010 | *ACS3-1* | GAAGCCGAGATGGAACTCTGGAAG | TTGTTGTTGGTGGTGGTTGAGGAG |
| VIT_200s1437g00010 | *ACS3-2* | ATGGAGGTTCTAATGGACAGG | CTGCCACATCGTTGGAGTAAA |
| VIT_211s0016g02560 | *ACS7* | ATCCTTTAGGCACAACCATC | CTTTATTATTGCGGGCTTCG |
| VIT_200s2086g00010 | *ACO1* | TAAGCCTAGCTACTGATGAGTG | CATGGTACATCAAGCCTAAG |
| VIT_214s0081g00730 | *ERF1B* | GGGTTCACTGGCTGTTCTCA | GCTCCTCCAAGTAATCTGTC |
| VIT_215s0021g01610 | *ERF2* | GCGTATTCCACCACTACTCCC | ACTCCTCTGTACCTCCTCCACTC |
| VIT_200s0184g00110 | *IAA12* | GCTTTTAGTCATCAACCAGTG | CATATTCGACACATGGAATG |
| VIT_203s0091g00310 | *GH3.1* | CCACAGACCAATCAAATAGTG | TCATTTGGACACTCATCCTG |
| VIT_209s0054g00725 | *SAUR32* | TGATGTATGGTTTGTGATTCTC | CTAATAGCCTCCAAGTTTGG |
| VIT_217s0000g00941 | *SAUR40* | AGGTGAATTGAGCATCGACTG | CACCAGCTTCGTCCCTGC |
| VIT_218s0166g00050 | *Protein kinase 1* | GTTTGTGTTGTGCCGATC | ATTAAAGACAGAATGGGAGG |
| VIT_204s0044g00840 | *LECRK3* | GAAGAGGTGGACAAACGAGA | GTACTGGGAGGTACAGGGAT |
| VIT_214s0108g00340 | *MIK2-1* | ACTGTGGACTCAATGGCAGCATC | CCAACTGAGTGAGGTTAGCCAAGG |
| VIT_218s0072g00990 | *PXC3* | TGAACAACACAGCACAGCGAGAC | GCACGACACTAACACAGCCTAGAG |
| VIT_211s0037g00660 | *MIK2-2* | TGGCACTGGAGGACATGGAGATG | ACAATGTTGCGGTGCCGTATCG |
| VIT_216s0050g01780 | *MAKR5* | GAACAGCAACAAGAGCGGAGAGG | AACAGCGAGATCGGAGACTGAGG |
| VIT_214s0066g02660 | *Protein kinase 2* | TGGCTGAGGCTGACGCTCTG | CCGACCAACTGCAATGGTTAGAGG |

**Table S1** Primer sequences for real-time quantitative RT-PCR.

Gene ID is derived from the grape genome (http://genomes.cribi.unipd.it/grape/)
